# Association of juvenile idiopathic arthritis with *PTPN22* rs2476601 is specific to females in a Greek population

**DOI:** 10.1186/s12969-016-0087-3

**Published:** 2016-04-23

**Authors:** G. N. Goulielmos, R. C. Chiaroni-Clarke, D. G. Dimopoulou, M. I. Zervou, M. Trachana, P. Pratsidou-Gertsi, A. Garyfallos, J. A. Ellis

**Affiliations:** Laboratory of Molecular Medicine and Human Genetics, Department of Medicine, Medical School of Crete, Heraklion, Greece; Genes, Environment and Complex Disease, Murdoch Childrens Research Institute, Parkville, Victoria 3052 Australia; Department of Paediatrics, University of Melbourne, Parkville, Victoria Australia; Rheumatology Unit, 4th Department of Internal Medicine, Aristotle University of Thessaloniki, Hippocration Hospital, Thessaloniki, Greece; Pediatric Immunology and Rheumatology Referral Center, 1st Department of Pediatrics, Aristotle University, Thessaloniki, Greece

**Keywords:** Juvenile Idiopathic Arthritis (JIA), Genetic association, Gene polymorphism, Protein tyrosine phosphatase nonreceptor 22 (*PTPN22*) gene, Sex

## Abstract

**Background:**

Juvenile idiopathic arthritis (JIA) is an autoimmune disease characterized by persistent chronic arthritis. Disease risk is believed to be influenced by both genetic and environmental factors. It is well established that the *PTPN22* single nucleotide polymorphism (SNP) rs2476601 is associated with JIA susceptibility. It was recently reported in an Australian study that this association is restricted to females and is not observed in males. A significant source of inconsistency amongst the literature on autoimmune disease susceptibility genes stems from an inability to replicate genetic findings across different racial or ethnic groups. We therefore attempted to generate further evidence of the female-specific association of rs2476601 in a homogeneous Greek population.

**Findings:**

We genotyped rs2476601 in 128 Caucasian JIA patients (70.3 % female) and 221 healthy controls (28.1 % female) from Northern Greece. Overall, *PTPN22* was associated with increased risk of JIA in this Greek sample (OR = 2.3, 95 % CI 1.1 – 5.1, *p* = 0.038). Sex-stratified analyses showed that, once again, the risk association was restricted to females (Female: OR = 19.9, 95 % CI 1.2 – 342, *p* = 0.0016; Male: OR = 1.1, 95 % CI 0.3 – 3.1, *p* = 0.94) supporting the prior findings.

**Conclusions:**

Our data demonstrates that this sex-specific pattern of association is broadly applicable to different populations, and provides further impetus to undertake mechanistic studies to understand the impact of sex on *PTPN22* in JIA.

## Findings

### Introduction

Autoimmunity is believed to result from a combination of genetic factors and environmental triggers and one feature that is shared among many autoimmune disorders, including juvenile idiopathic arthritis (JIA), is the increased proportion of females that develop the disorder. The reason for the observed sex bias remains elusive, and relatively few studies have comprehensively investigated this phenomenon from a mechanistic perspective, especially in JIA.

JIA is the most common chronic arthritis of childhood [1] and is associated with significant morbidity. JIA is an umbrella term that encompasses a heterogeneous group of disorders (subtypes) all characterized by prolonged synovial inflammation that may cause destructive damage to joint structures [2]. The subtypes oligoarthritis (both persistent and extended) and rheumatoid factor (RF)–negative polyarthritis are considered the most homogeneous subtypes, with shared phenotypic features, while systemic JIA has a more distinct phenotype resembling an autoinflammatory syndrome [3]. Interestingly, not all JIA subtypes are more common in females, but the oligoarticular and polyarticular subtypes, which constitute around 70 % of all cases, occur 2-3 times more frequently in girls [1].

Accumulated evidence indicates that ethnic heterogeneity may complicate genetic association studies in rheumatic diseases [4–7]. There is substantial ethnic variability in allele frequencies of common single nucleotide polymorphisms (SNPs) that have been catalogued since the completion of the human genome sequence. Polymorphisms in various genes associated with rheumatic diseases have been reported to vary substantially by allele frequency in different ethnic groups [8, 9].

A non-synonymous SNP, rs2476601 (also referred to as C1858T or R620W), located in the gene encoding protein tyrosine phosphatase nonreceptor 22 (*PTPN22*) has been repeatedly associated with a wide range of autoimmune diseases, including JIA [10]. While the data are inconsistent, multiple recent studies have suggested that this SNP may be associated with disease in a sex-specific manner. For example, in type 1 diabetes, rs2476601 appears only related to disease risk in females [11–13]. The same SNP was found to be associated only in males with psoriatic arthritis (PsA) [14] and rheumatoid arthritis (RA) [15].

In relation to JIA, it was recently reported that *PTPN22* rs2476601 is associated with JIA only in females in an Australian case-control sample. The finding was replicated in a second case-control sample from the US and Norway [16]. This present study aimed to investigate whether the female-specific association can also be observed in further ethnic groups by performing sex stratified association analysis in a Greek JIA case-control sample.

### Materials and methods

#### Patient population and study design

In this case-control association study, one hundred and twenty eight JIA patients (70.3 % female), followed in the Rheumatology Units of the 4^th^ Medical Department of Internal Medicine and the 1^st^ Medical Department of Pediatrics of Aristotelian University of Thessaloniki Hospital (Northern Greece) were available for analysis. Only patients who fulfilled the 2001 revised International League of Associations for Rheumatology (ILAR) criteria for JIA and gave their informed consent were eligible for the study. Cases were of mixed ILAR subtype, as detailed in [6]. Two hundred and eleven controls (28.1 % female) were also available. Controls consisted of healthy subjects without autoimmune or chronic inflammatory disease, or an acute or chronic infectious disease; and were recruited from the Transfusion Medicine Department of Hippokration Hospital of Thessaloniki (Greece). Cases and controls, including criteria for study inclusion, are described in further detail in [6]. All patients were of self- or parent-reported Greek origin. The study was approved by the local Ethics Committee for medical research (General Assembly of the Medical School of the Aristotle University of Thessaloniki and Hippokration Hospital, G.A. 06/07-11-2007) and was carried out in compliance with the declaration of Helsinki.

#### Genotyping

Genomic DNA was isolated from peripheral blood leukocytes by using the commercial kit PUREGENE (Gentra Systems, Minneapolis, MN) according to the manufacturer’s instructions and genotyping was performed for the *PTPN22* rs2476601 (*Xcm*I) polymorphism by restriction fragment length polymorphism (RFLP) analysis as described previously [4].

#### Statistical analysis

Initial statistical analyses were performed using GraphPad Prism statistical program (GraphPad Software, San Diego, CA). All cases and controls used in the analysis were unrelated. The genetic variant under investigation was evaluated for deviation from Hardy–Weinberg equilibrium (HWE) by comparing observed and expected genotype frequencies by means of chi-squared (x^2^) test both in case and control groups. The HWE test was performed in combination with an analysis for ascertainment bias for dominant/recessive models (due to biological or technical causes) by using the program "Calculate" (Copyright TRG, SR, INMD, 2008). Further statistical analyses were undertaken using Stata v14 (StataCorp, College Station, TX, USA). Logistic regression was used to generate ORs and 95 % CIs for the association of rs2476601 with JIA. For analyses where logistic regression could not be performed because of the absence of the minor allele in any one group, we used the Gart method [17] with a continuity correction delta = 0.5 (also known as the Woolf method) to estimate ORs and 95 % CIs, and Fisher’s exact test to determine the *P*-value. We also performed a ‘pooled control’ analysis (female cases compared to all controls and male cases combined), following past work on *PTPN22* and JIA in other populations [16]. A *P* value less than 0.05 was considered significant.

### Results and discussion

Allele frequencies of the *PTPN22* rs2476601 C/T SNP in males and females are shown in Table 1. No deviation from HWE was observed in either cases or controls. Overall (both sexes together), we observed a significant association of rs2476601 with JIA in this Greek population (OR = 2.3, 95 % CI 1.1 – 5.1, *p* = 0.038) in a direction consistent with previous findings. When stratified by sex, we observed association of rs2476601 with increased risk of JIA in females (OR = 19.9, 95 % CI 1.2 – 342, *p* = 0.0016). The wide confidence interval reflects the small sample size, and the fact that no minor alleles were observed in female controls. The pooled-control analysis, which hypothesizes that male JIA risk is not affected by rs2476601 and therefore male cases are essentially controls, further supported an association of rs2476601 with female JIA (OR = 2.5, 95 % CI 1.1 – 5.6, *p* = 0.025). In males, no association between rs2476601 and JIA was observed (OR = 1.1, 95 % CI 0.3 – 3.1, *p* = 0.94). Exclusion of systemic JIA cases did not materially alter the findings. This sex-specific pattern of association, including the direction of effect, is consistent with the prior study conducted in Australian, US and Norwegian samples [16]. Given that the sex bias differs by JIA subtype, ideally we would have liked to test for these associations in individual subtypes; however, small sample size (for details see [6]) prevented us from performing these analyses with any statistical certainty.

**Table 1:** Association of *PTPN22* rs2476601 with JIA in the Greek population, stratified by sex

	Case/Control N	MAF (case/control)	OR (95 % CI)
All	128/221	0.059/0.027	2.3 (1.1 – 5.1) *p* = 0.038
Female	90/61	0.066/0	19.9 (1.2 – 342) *p* = 0.0016*
Male	38/160	0.039/0.038	1.1 (0.3 – 3.1) *p* = 0.94

*Cannot perform logistic regression as no minor allele present in cases in this subset. OR calculated using Gart method (continuity correction delta =0.5). *P*-value calculated by Fisher’s exact test.

*MAF* minor allele frequency

Over the last decade, several studies have investigated candidate genes to discover or confirm associations with JIA phenotypes (e.g. [18]). Importantly, associations of gene variants with JIA have not always replicated when tested in different populations [19]. This is the first study examining the association of *PTPN22* rs2476601 specifically with JIA in female patients in a population from Greece.

*PTPN22* contributes to the modulation of negative T cell selection in the thymus and downregulation of autoreactive T cells in the periphery [20]. It has been demonstrated that PTPN22 increases phosphatase activity when the minor ‘T’ allele of rs2476601 is present [21]. As a consequence, this gain of function associated with the ‘T’ allele may suppresses T cell signaling more efficiently and lead to a failure in apoptosis of autoreactive T cells and to insufficient activity of regulatory T cells [22]. Taking into consideration that females have higher numbers of CD4 lymphocytes than males and higher levels of Th1 cytokines [23], the observed role of the *PTPN22* polymorphism may be mainly relevant to females [13].

*PTPN22* rs2476601 shows a wide variation in “T” allele frequencies among different ethnic populations, with a North–South gradient in Europe ranging between 15.5 % (north) and 2.1 % (south) [8]. Lee et al*.* [24] reported that this allele frequency ranged between 15.8 and 8.7 % for JIA patients and 11.6 and 7.8 % for controls. Accordingly, the observed presence of the rs2476601 minor allele in Greece was lower than that in North or Central European populations but similar to that in a Turkish population, which is geographically closer to Greece [5]. However, despite this, the SNP has shown an association with JIA, specifically in females, in this Greek case-control sample [6].

In conclusion, our results provide additional evidence for a female-specific association between the rs2476601 *PTPN22* SNP and the risk of JIA by independent replication in a Greek population, demonstrating the broader applicability of this finding across various ethnic groups. This locus is of particular interest in this context given that the genetic variant under investigation occurs at different frequency in the Greek population compared to other European populations. The importance of comparative studies in populations of different ethnic and/or racial origins is high in any attempt to conclusively define the genetic architecture of JIA and the magnitude of the effects of specific risk alleles in different populations.
